# Supplementary figures and images for: Conserved and novel enhancers in the Aedes aegypti single-minded locus recapitulate embryonic ventral midline gene expression
Source: PLoS Genet. 2024 Apr 29;20(4):e1010891. doi: 10.1371/journal.pgen.1010891 (PMC11081499; doi:10.1371/journal.pgen.1010891)

Schember et al. 2024. Supplementary Figure S1

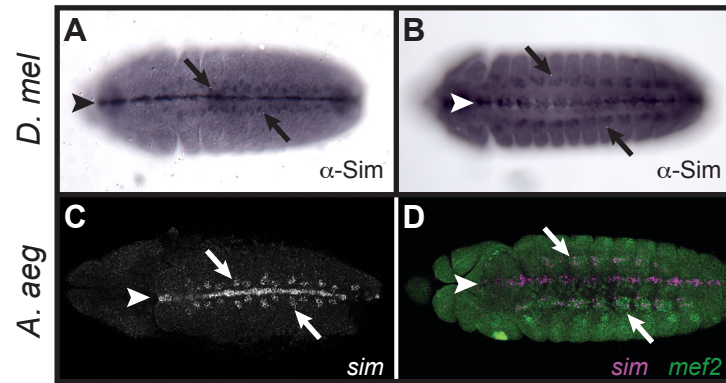

Supplement: S1 Fig — (A, B) Drosophila embryos stained with anti-Sim antibodies at stages 11 and 14, respectively, show Sim expression in both the midline (arrowheads) and muscle (arrows). (C, D) Similar expression is detected by HCR in A. aegypti embryos at analogous stages. The embryo in D is co-labeled with HCR for sim (magenta) and the muscle-specific gene mef2 (green). (PDF) [file pgen.1010891.s001.pdf]

Supplemental Figure 2

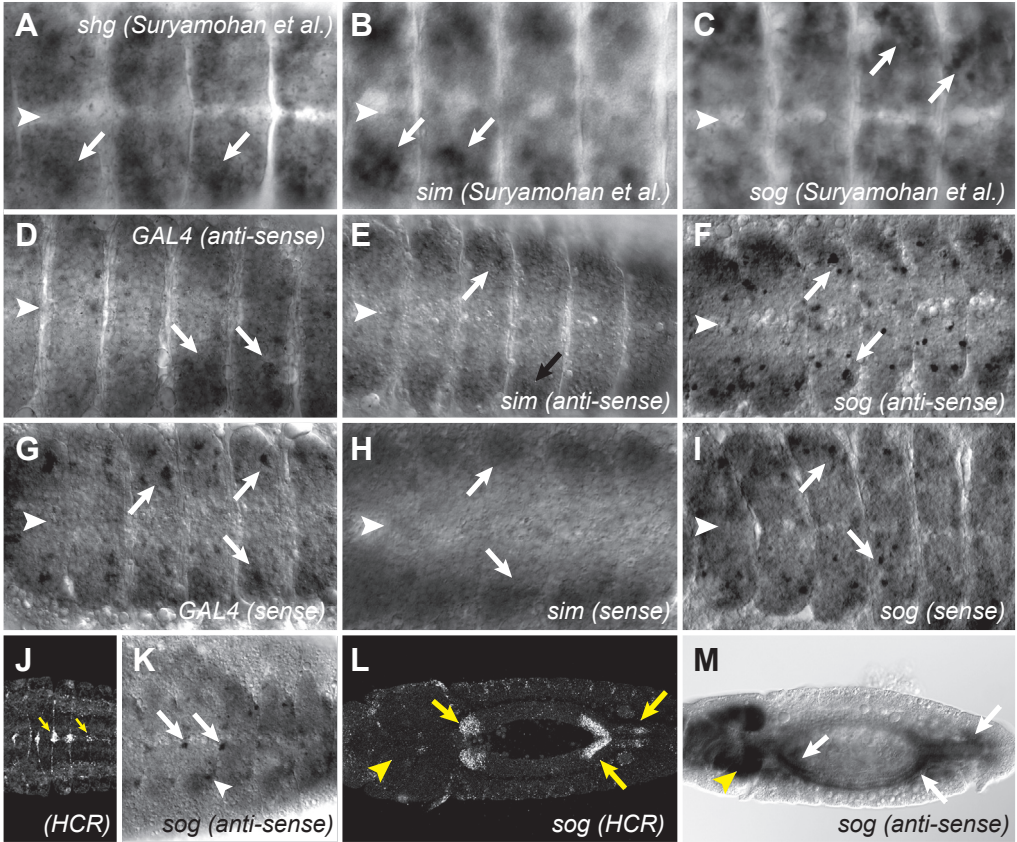

Supplement: S2 Fig — (A-C) In situ hybridization results reported by Suryamohan et al. [18] for the A. aegypti orthologs of Drosophila (A) shg, (B) sim, and (C) sog. All three genes appear to be absent from the midline (arrowheads) but expressed in lateral regions of the nerve cord (arrows). (D-F) New in situ hybridization results using anti-sense probes for yeast Gal4 (D), and A. aegypti sim (E) and sog (F). Note again the overall absence of expression in the midline (arrowheads) and presence in lateral nerve cord regions (arrows). (G-I) In situ hybridization using control (sense) probes for the same sequences as D-F. Note the qualitatively similar appearance of limited midline but prominent lateral expression. (J, L) Results for HCR against A. aegypti sog compared to (K, M) standard in situ hybridization results suggest that the standard in situ method does work in some tissues, but is heavily artifact prone in others. Although among the positive-appearing cells in panel K are midline cells morphologically similar to the dorsal median cells of Drosophila (arrows), which are clearly positive by HCR in J (arrows), there are also lateral cells (arrowhead) with no corresponding HCR signal. Similar positive signal can be observed in the anterior and posterior midgut in L and M (arrows), but note the apparent artifact of strong signal in the brain in M whereas there is no corresponding expression observed by HCR in L (arrowheads). (PDF) [file pgen.1010891.s002.pdf]

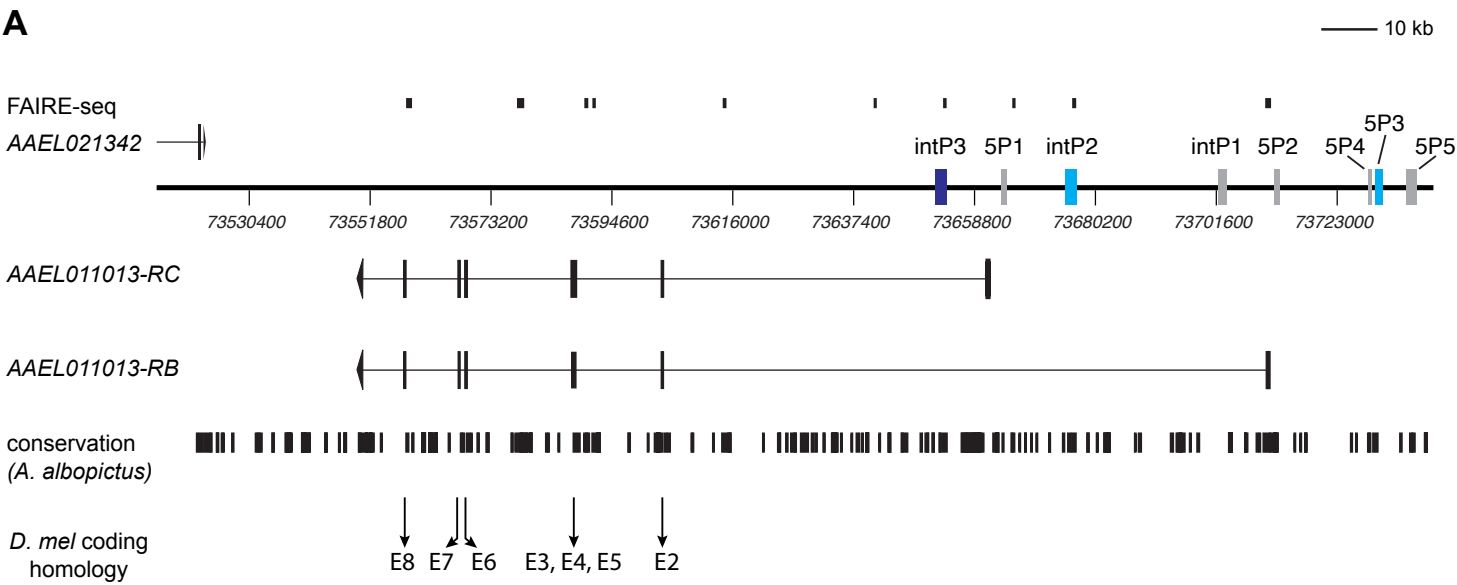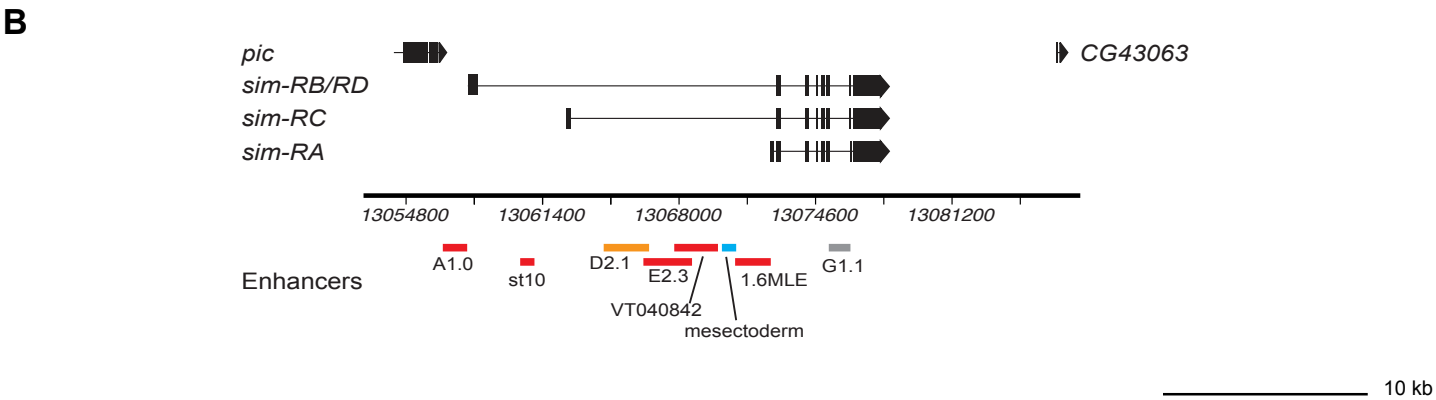

Supplement: S3 Fig — Note that the two maps are at different scales. (A) The A. aegypti sim locus (Vectorbase gene AAEL011013). The two annotated transcripts are shown along with positions of the sequences tested for enhancer activity in this paper. Sequences with no embryonic reporter gene activity are shown in gray, midline activity in cyan, and ectopic activity in dark blue. Positions of FAIRE peaks (from [46]), conservation with A. albopictus, and exon conservation with D. melanogaster are shown. Conservation with A. albopictus was assessed by Blast2Seq run on the NCBI Blast server [70] using word size = 11, match/mismatch = 2,-3 and gapcosts = 5,2. (B) The D. melanogaster sim locus showing the annotated transcripts and a subset of the known sim enhancers. Enhancers with midline activity are in red, early (mesectoderm stage) activity in cyan, and weak midline activity in orange. Sequence G1.1, shown in gray, has conflicting reports in the literature about its midline activity but in our hands lacks midline expression. (PDF) [file pgen.1010891.s003.pdf]

Supplementary Figure 4

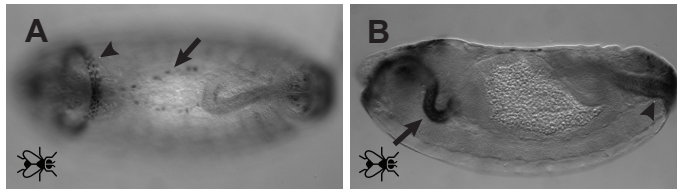

Supplement: S4 Fig — All observed intP3 activity is ectopic with respect to sim. (A) Dorsal view of a stage 14 embryo showing reporter gene expression in the developing dorsal vessel (arrowhead) and in a ring of cells in the anterior region of the embryo, provisionally identified as atrium precursors. (B) Sagittal view of a stage 15 embryo showing prominent expression in the foregut (arrow) as well as weaker expression in the hindgut (arrowhead). (PDF) [file pgen.1010891.s004.pdf]

Schember et al. 2024. Supplemental Figure S5

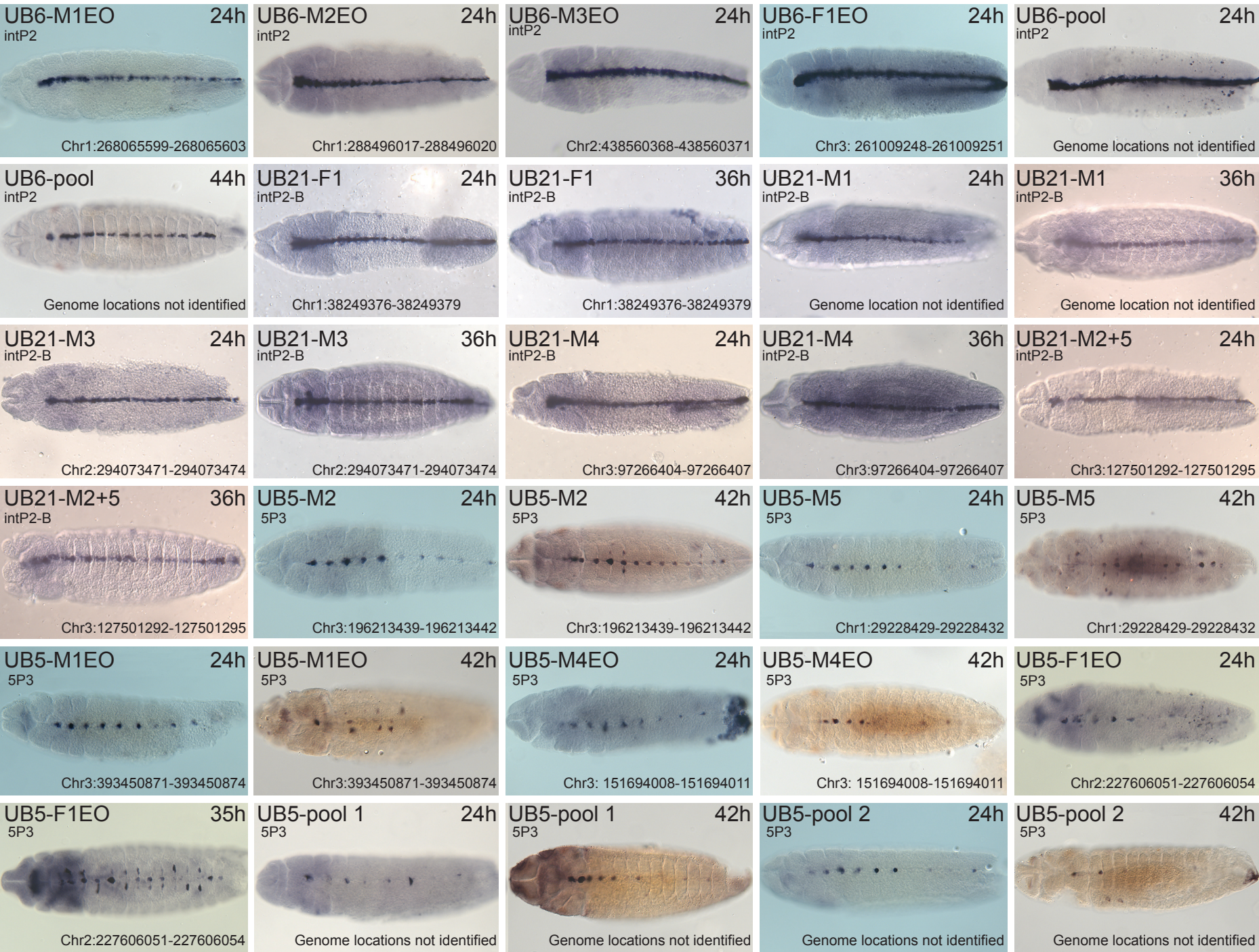

Supplement: S5 Fig — Immunohistochemical staining of the eGFP reporter in additional lines of transgenic A. aegypti for the intP2, intP2B, and 5P3 CRMs. No qualitative differences in expression pattern were observed among the multiple independent lines for each reporter construct. All embryos are oriented ventral side up with anterior to the left. (PDF) [file pgen.1010891.s005.pdf]

Schember et al. 2024 Supplemental Figure S7

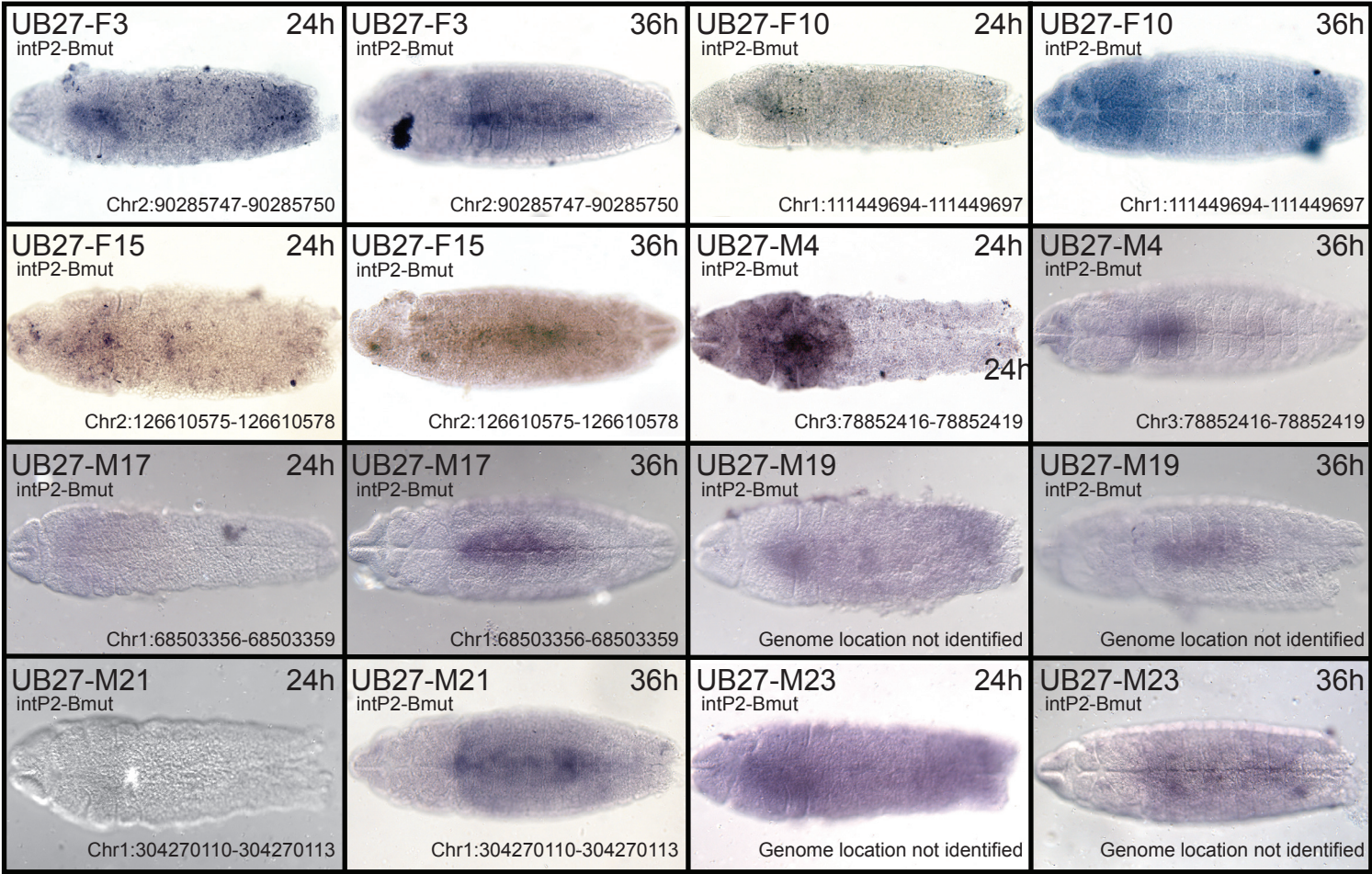

Supplement: S7 Fig — Immunohistochemical staining of the eGFP reporter in seven additional lines of transgenic A. aegypti for the intP2Bmut CRM (line UB27-F15 is pictured in Fig 2L and 2M). No qualitative differences in expression pattern were observed among the multiple independent lines. All embryos are oriented ventral side up with anterior to the left. (PDF) [file pgen.1010891.s007.pdf]

Schember et al. 2024. Supplemental Figure S8

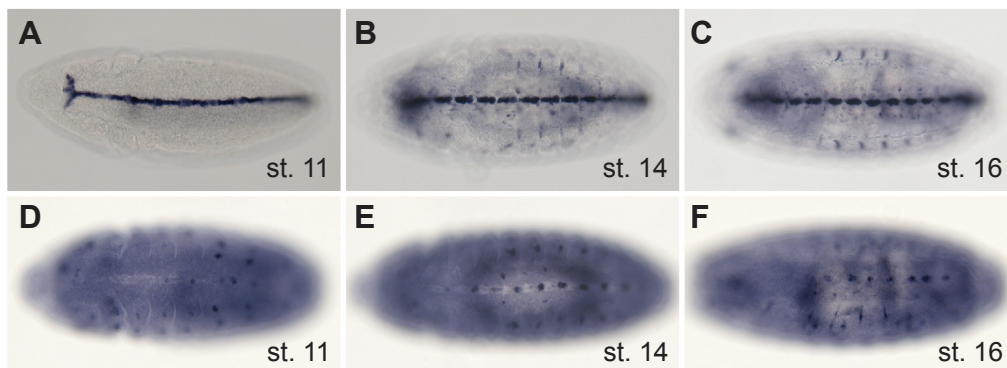

Supplement: S8 Fig — (A-C) The A. albopictus intP2B CRM is active in the expected pattern in Drosophila embryos at stages 11, 14, and 16, respectively. (D-F) The A. albopictus 5P3 CRM is active in the expected pattern in Drosophila embryos at stages 11, 14, and 16, respectively. Some segments are out of focus. All embryos are oriented ventral side up with anterior to the left. (PDF) [file pgen.1010891.s008.pdf]

Schember et al. 2024 Supplemental Figure S9

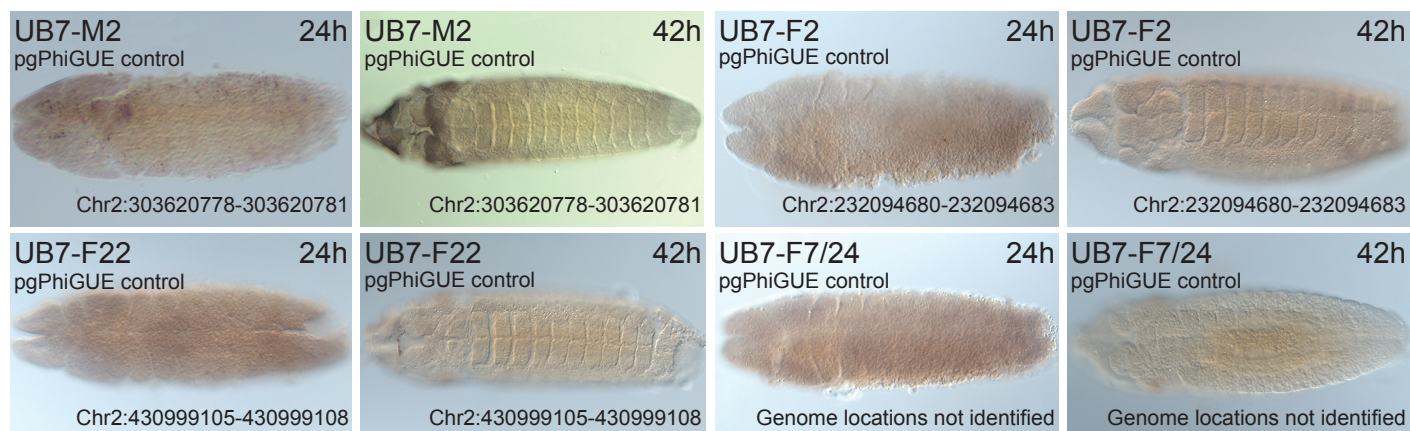

Supplement: S9 Fig — Immunohistochemical staining of the eGFP reporter for a negative control reporter (pgPhiGUE_LANDR, courtesy of Kevin Deem) demonstrating the absence of vector-dependent reporter gene activity. All embryos are oriented ventral side up with anterior to the left. (PDF) [file pgen.1010891.s009.pdf]

Figure S10

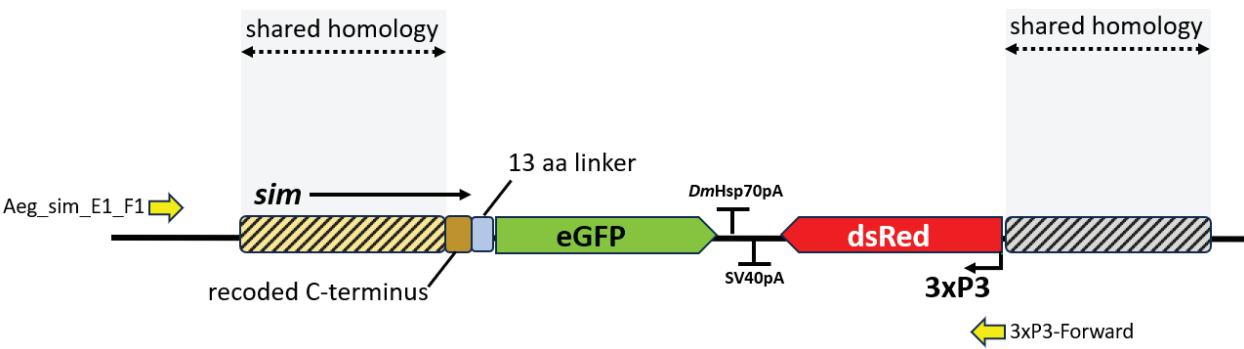

Supplement: S10 Fig — Sequencing through the region with primers Aeg_sim_E1_F1 and 3xP3-Forward confirmed the correct integration. (PDF) [file pgen.1010891.s010.pdf]
