# Supplementary material for: Conserved and novel enhancers in the Aedes aegypti single-minded locus recapitulate embryonic ventral midline gene expression
Source: PLoS Genet. 2024 Apr 29;20(4):e1010891. doi: 10.1371/journal.pgen.1010891 (PMC11081499; doi:10.1371/journal.pgen.1010891)
Supplement: S6 Fig — Sequences of known D. melanogaster sim enhancers were obtained from REDfly [71] and analyzed for a subset of putative transcription factor binding sites using consensus sequences as described in Pearson et al. [25]. sim_minimalMLE is a subfragement of sim_1.6MLE (S3B Fig). The 2.3 kb sim_E2.3 sequence is not included. A. aegypti intP2B is from the current study. The scale along the top indicates size in basepairs. (PDF) [file pgen.1010891.s006.pdf]

Supplemental Figure 6

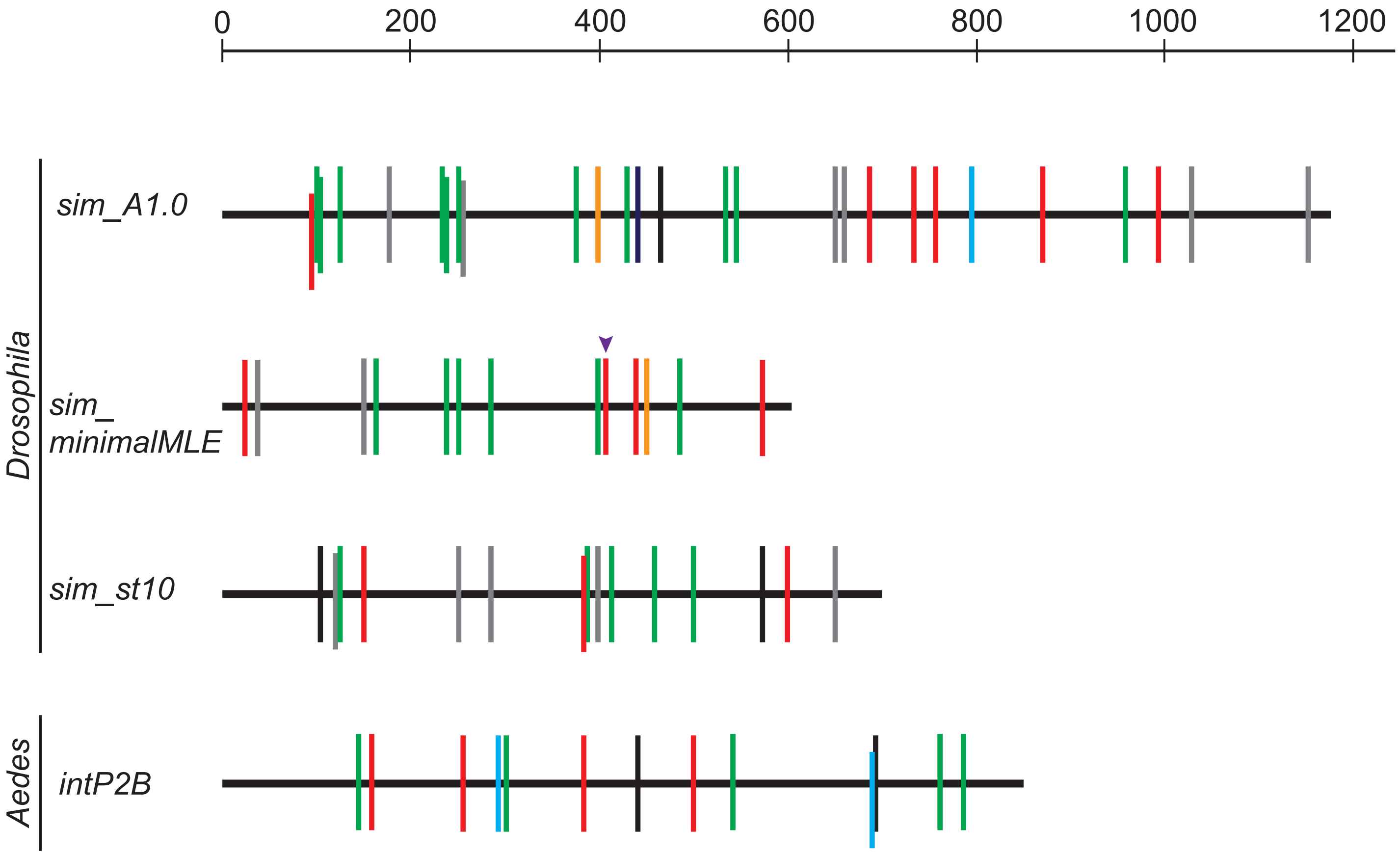

RED: CME (Sim:Tgo)
GREEN: Vvl
BLACK: Twi
GRAY: E-box (not Twi preferred)
PURPLE arrowhead: MotifA
ORANGE: Zld
CYAN: Su(H)
