# Supplementary material for: Conserved and novel enhancers in the Aedes aegypti single-minded locus recapitulate embryonic ventral midline gene expression
Source: PLoS Genet. 2024 Apr 29;20(4):e1010891. doi: 10.1371/journal.pgen.1010891 (PMC11081499; doi:10.1371/journal.pgen.1010891)
Supplement: S1 Text — Tables A-C. (DOCX) [file pgen.1010891.s011.docx]

**Table A:** Primers for transgenic fly and mosquito lines

| **Fragment** | **Vector^a^** | **Forward primer^b^** | **Reverse Primer^c^** |
| --- | --- | --- | --- |
| *intP1* | pLacZattB | tcgcaggcctcgcaccggtg*aattc*ACTATGTCGATAACTGGTAC | ccggcgctctagaggtaccc*tcgag*CTTAAGGTCAAATACAAATGG |
| *intP2* | pLacZattB &  pgPhiGUE | tcgcaggcctcgcaccggtg*aattc*CAATTTAGTAGTTTTTTTTTGCAATTTC | ccggcgctctagaggtaccc*tcgag*GTGGTAAAAATCAATAGATTCC |
| *intP2A* | pLacZattB | tcgcaggcctcgcaccggtg*aattc*CCGTGCGTCCGTTAACAAATC | ccggcgctctagaggtaccc*tcgag*CGTATCTGGACCACTGTG |
| *intP2B* | pLacZattB | tcgcaggcctcgcaccggt*gaattc*GCTGCTAAAATGCCGAGTC | ccggcgctctagaggtacc*ctcgag*TATTACCCCCTCCCCTCG |
| *intP3* | pLacZattB | tcgcaggcctcgcaccggtg*aattc*TGAAAATATTTGGAAACGACTTAC | ccggcgctctagaggtaccc*tcgag*TCTCAATTATACGCGGCG |
| *5P1* | pLacZattB | tcgcaggcctcgcaccggtg*aattc*ATATGCAAATCATATTCTGATAAAATTTTATTATATTT | ccggcgctctagaggtaccc*tcgag*ACCAGAATGATAATTAAAATATAAATGTC |
| *5P2* | pLacZattB | tcgcaggcctcgcaccggtg*aattc*CTCATCATGAAATTTCCAGATAG | ccggcgctctagaggtaccc*tcgag*TATTCTACTAACACCAAACTAG |
| *5P3* | pLacZattB &  pgPhiGUE | tcgcaggcctcgcaccggtg*aattc*ATTAAATAAACTTTACCGAATGATC | ccggcgctctagaggtaccc*tcgag*AATAAAAATGGCGACCGATG |
| *5P3A* | pLacZattB | tcgcaggcctcgcaccggtg*aattc*GCGCCAAGCCAAATTTAC | ccggcgctctagaggtaccc*tcgag*CACCGTGCCAGAGAACATC |
| *5P3B* | pLacZattB | tcgcaggcctcgcaccggtg*aattc*AAGCGAAAGGGTGTTCAAG | ccggcgctctagaggtaccc*tcgag*GGCAATGGAGGAGCAATG |
| *5P3D* | pLacZattB | tcgcaggcctcgcaccggtg*aattc*TTCCATTCAACGTTTGCC | ccggcgctctagaggtaccc*tcgag*AGTCTATGGTAGCACTGC |
| *5P3E* | pLacZattB | tcgcaggcctcgcaccggtg*aattc*TGAGTTCTAAGAGCATGTTCC | ccggcgctctagaggtaccc*tcgag*TAAATCGCCAGGTGAGCC |
| *5P3F* | pLacZattB | tcgcaggcctcgcaccggtg*aattc*TTCAACGTTTGCCCGGAAAC | ccggcgctctagaggtaccc*tcgag*ATGGCGACCGATGGTTGTTAC |
| *5P3G* | pLacZattB | tcgcaggcctcgcaccggtg*aattc*TCCTTTCAAGTCCTAAAAGAAAAGAACATTGTTAC | ccggcgctctagaggtaccc*tcgag*CGGTTTCCGGGCAAACGTTG |
| *5P3H* | pLacZattB | tcgcaggcctcgcaccggtg*aattc*ACCCTTAAATAAATCCCCAATTTG | ccggcgctctagaggtaccc*tcgag*TCTTTTCTTTTAGGACTTGAAAG |
| *5P3I* | pLacZattB | tcgcaggcctcgcaccggtg*aattc*TGAGTTCTAAGAGCATGTTCC | ccggcgctctagaggtaccc*tcgag*GCAAATTGGGGATTTATTTAAG |
| *5P4* | pLacZattB | tcgcaggcctcgcaccggtg*aattc*TGACATTTGTAGCATTGTCAC | ccggcgctctagaggtaccc*tcgag*ATAGCAGGACCGTACCAG |
| *5P5* | pLacZattB | tcgcaggcctcgcaccggtg*aattc*ATGAAGCAAGCACCTCGG | ccggcgctctagaggtaccc*tcgag*CCGACCTTCCACAGCTATC |

^a^ The *pLacZattB* vector was used to make transgenic *D. melanogaster*, while the *pgPhiGUE* vector was used to make transgenic *A. aegypti*

^b^ Lowercase letters are specific to *pattBLacZ*, italics indicate the restriction site for *EcoRI*, and uppercase letters are specific to the amplified *A. aegypti* sequence

^c^ Lowercase letters are specific to *pattBLacZ*, italicized indicate the restriction site for *XhoI*, and uppercase letters are specific to the amplified *A. aegypti* sequence

**Table B.** Genomic insertion sites for the transgenic *Ae. aegypti* lines

| **Construct** | **Line prefix** | **Line** | **Genomic insertion site (LVP genome version: AaegL5.3)** |
| --- | --- | --- | --- |
| *5P3* | UB5 | M1EO | Chr3:393450871-393450874 |
|  |  | M5 | Chr1:29228429-29228432 |
|  |  | F1EO | Chr2:227606051-227606054 |
|  |  | M2 | Chr3:196213439-196213442 |
|  |  | M4EO | Chr3:151694008-151694011 |
|  |  | pool #1 | pooled line* |
|  |  | pool #2 | pooled line* |
| *intP2* | UB6 | M1EO | Chr1:268065599-268065603 |
|  |  | M2EO | Chr1:288496017-288496020 |
|  |  | M3EO | Chr2:438560368-438560371 |
|  |  | F1EO | Chr3:261009248-261009251 |
|  |  | pool #1 | pooled line* |
| *pgPhiGUE_LANDR* | UB7 | F2 | Chr2:232094680-232094683 |
|  |  | M2 | Chr2:303620778-303620781 |
|  |  | F22 | Chr2:430999105-430999108 |
|  |  | F7, F24 | pooled line* |
| *intP2B* | UB21 | F1 | Chr1:38249376-38249379 |
|  |  | M1 | Insertion not identified |
|  |  | M2+5 | Chr3:127501292-127501295 |
|  |  | M3 | Chr2:294073471-294073474 |
|  |  | M4 | Chr3:97266404-97266407 |
| *intP2Bmut* | UB27 | F3 | Chr2:90285747-90285750 |
|  |  | F10 | Chr1:111449694-111449697 |
|  |  | F15 | Chr2:126610575-126610578 |
|  |  | M4 | Chr3:78852416-78852419 |
|  |  | M17 | Chr1:68503356-68503359 |
|  |  | M19 | Insertion not identified |
|  |  | M21 | Chr1:304270110-304270113 |
|  |  | M23 | Insertion not identified |

*Genomic insertion sites were not determined for pooled samples with multiple piggyBac insertion sites.

**Table C.** Primers and gBlocks used to generate the *sim*::eGFP fusion line of *Ae. aegypti.*

| Primer/gBlock | Sequence (5’ -> 3’) |
| --- | --- |
| *sim-eGFP-don-MT* | caattgggcccGATCCaacaccagcagccggggtacgatgagataaatCAAcacttcggtctgaactgtttcaacaataataataccagcaacggcaaTggcgccattcctgccaacggtcatttgaatggtggtctcaacagtcacactctacatcaccagcacagtgccaacagcaataacggaagcaacagtagcagcgccagtgctgccaacagtaataacaacaatcacgacaatggcttcatggatgagttcaaagtaagccaacagcatcaccatcatccccagcagcagcaacagcatgtcgtccctcagcagcaccatcatccgcaccaccaacaacatcaccaccacagtcacaacggggcagggggaggacctcagtacaccagcgtgattgtggagccgCAAaattaccacctaccagtaactcagaatgagttcgttcacggatcggctggatcggctgctggatcgggagaattccaattctcgagctatcatctgccggtcacgcaaaacgaatttgtgcattaggcgccgccattttccccctcctttccctccgggaaggagtttatgcatcgtcagtgggcaaaaactgactgaaagcagcaaagaattAagcagtttgtgctgccgttgattgtgaataacgagttagactatttttgaaaaggaaaacctctaaagggaatcgattgatactagtttcaaccattgcgagttgatctgaggggctcatttagtcccaaattcccttgcattttagtagtttttctagaacttattatagttttgcgtcttgtttcatgtgattattgtttcgaaatgttttcatattgtacagagatacagtgctggacattattatgtaatGtcgtttttcacacaaaatccacatctccatacattcaacgcactgtgcggcTGTGCTGTGCGACTCCGTCGAGTCGACCAACATAGTTGAAACAAATTGAATATTTAATTGATCGTTATAGGAATGGTGTTAGATGAGTCATCCTTTACAGTAAGCACATACAGTATTATAATTGAAGATCGTCGGCAGATAGGTGTGTAGGGTAGAGTATCAGCAATAAGTTGGGACGTTTGACTTTTTGTAGGTAGACAAAAACTAAACtttttttCGCTTCTCTATGTGTGCCCCTGGGTAGCGTTCCGTTCCGATTGGGGTGCGAACGAATGAAATCGCCCATCGAGTTGATACGTCCATCCATCGCTAGAACCGCGTTCGCTGTAaAAGACTATATAAGAGCAGAGGCAAGAGTAGTGAAATaccggcagatgatagttttgGTTTCAGAGCTATGCTGGAAACAGCATAGCAAGTTGAAATAAGGCTAGTCCGTTATCAACTTGAAAAAGTGGCACCGAGTCGGTGCTTTTTTTTTgctagc |
| *eGFP-fus-EcoRI-F* | caattGAATTCatggtgagcaagggcgag |
| *3xP3-fus-XhoI-R* | caattCTCGAGtgatcgcacggttcccac |
| *Aeg_sim_E7_F1* | CCAGCAAACAAGAAGCGCACG |
| *3xP3-Forward* | aattcgagctcgcccgg |
